# Supplementary material for: Complexome profiling of the Chlamydomonas psb28 mutant reveals TEF5 as an early PSII assembly factor
Source: Plant Cell. 2025 Mar 17;37(6):koaf055. doi: 10.1093/plcell/koaf055 (PMC12164590; doi:10.1093/plcell/koaf055)
Supplement: koaf055_Supplementary_Data [file koaf055_supplementary_data.zip › psb28_interactive.zip!/plots/intensity/qualityPlots/peptides/PRA1_Cre01.g012500.t1.2.html]

### Cre01.g012500.t1.2

Trivial name: PRA1  
SeqCoverage [median]: 12.20  
Mass: 21.86 kDa  
  
 

| Sequence | MissedCleavage | Mass [Da] | wtIntensity | muIntensity | Score |
| --- | --- | --- | --- | --- | --- |
| LKEYASGVAR | 1 | 1092.59 | 0.00 | 3158.22 | 64.73 |
| NAFSKPGNLAEATSR | 1 | 1561.78 | 512.82 | 1107.68 | 79.32 |
|  |  |  | 512.8 | 4265.9 |  |
